# Familial health behavioral change in the context of adult treatment with obesity medications

**DOI:** 10.1016/j.obpill.2026.100305

**Published:** 2026-07-21

**Authors:** Treah Haggerty, Jennifer M. Ludrosky, Molly Powney, Reagan Sharp, Patricia Dekeseredy

**Affiliations:** a Department of Family and Community Medicine, West Virginia University School of Medicine, 6040 University Town Centre Drive, Morgantown, WV, 26501, USA; b Department of Behavioral Medicine & Psychiatry, Rockefeller Neurosciences Institute, West Virginia University School of Medicine, 930 Chestnut Ridge Rd, Morgantown, WV, 26508, USA; c West Virginia University School of Medicine, 1 Medical Center Drive, Morgantown, WV, 26506, USA; d WVU Department of Neurosurgery, West Virginia University School of Medicine, 1 Medical Center Drive, Morgantown, WV, 26506, USA

**Keywords:** Behavior change, Children, Family, Obesity treatment

## Abstract

**Background:**

Childhood obesity is a serious public health crisis in the United States, affecting millions of children annually and is associated with significant, long-term physical and psychological consequences. Family-based behavioral interventions have been shown to be effective in improving obesity-related outcomes in pediatric populations. However, there is limited insight into how adult participation in medical weight management programs may impact household health behaviors. This study explores parent perception of the impact of participation in weight management programs on child health behaviors.

**Methods:**

This is a qualitative study in which semi-structured interviews were conducted by trained personnel. Adult participants who were enrolled in a medical weight management clinic, had been prescribed obesity medication(s), and had at least one child aged 18 years old or younger living in the same household were included. Interviews were conducted between December 2024 and July 2025. Data analysis was guided through a thematic process; themes were discussed by the research group for consensus.

**Results:**

Ten interviews were conducted. Four themes emerged: (1) purposeful individual dietary choices, (2) acknowledgement of unintentional dietary changes in families secondary to program involvement, (3) consciousness of health behaviors across both individual and family levels, and (4) optimism regarding lifelong behavioral change despite concerns surrounding insurance coverage.

**Conclusion:**

Parents perceived that participation in medical weight management programs positively impacted household health behaviors. Participants across family systems identified both conscious and unconscious behavioral changes. These findings suggest that parent participation in weight loss programs may have broader implications beyond individual health benefits, and household health dynamics should be considered when managing pediatric obesity.

## Graphical abstract

**Figure:**
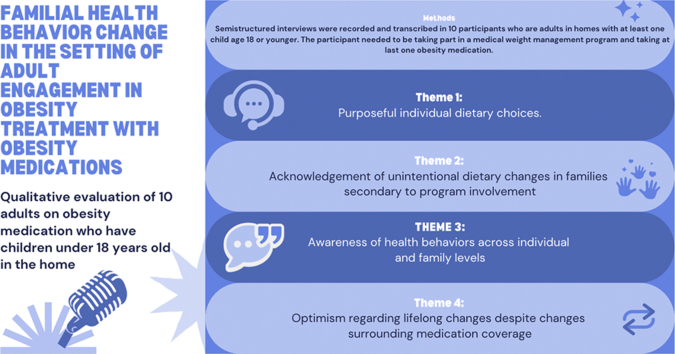

## 1 Background

Childhood overweight and obesity represent a significant health concern in the United States. Currently, almost 20% of children aged 2 through 19 years old have obesity [1]. Pediatric obesity is associated with increasing risks for various adverse physiological and psychological health consequences that persist into adulthood [2]. Childhood and adolescent obesity are associated with increased risk of premature death [3] (three times higher before age 30 compared to the general population), and nearly all organ systems are affected [4], and children with obesity have a higher likelihood of becoming adults with obesity. Obesity and the co-occurring chronic diseases also have a significant economic impact on the population: the global economic impact of overweight and obesity is expected to surpass $4 trillion by 2035, according to the 2023 World Obesity Atlas [5]. The health risks associated with childhood and adolescent obesity are modifiable with weight loss [4], suggesting that efforts to address obesity in childhood and adolescence have important and lifelong benefits.

Influences on the development of childhood obesity are complex, multifactorial, and include genetic, epigenetic, environmental [6], systemic [7], psychological [8], and behavioral [9] components. Efforts to address childhood and adolescent obesity frequently focus on the development and maintenance of beneficial health behaviors [10]. Families typically adopt lifestyle behaviors through modeling and through home environmental influences. “Parents are often considered a critical agent of change in behavioral lifestyle interventions, as they exercise significant control over children's eating and physical environment and, ultimately, behaviors.” [11] Children have a higher likelihood of adopting similar lifestyle behaviors to those of their parents for both movement and nutrition [12].

This positive effect of families as agents of change has been found to be true in health behavior change in relation to obesity treatment. Family-based interventions for childhood obesity are numerous and have been found to be effective [13]. Randomized controlled trials in primary care settings have demonstrated the significant benefit of family-based behavioral treatment on weight outcomes, both for the pediatric patient and siblings who did not receive direct treatment [14]. Family influences on child behavior are powerful; previous evidence suggests that parent-only interventions for childhood obesity treatment are comparable in effectiveness to parent-child interventions. Benefits to this approach could be increased scalability and program affordability.

The literature also shows that the influence of family behavior change may go beyond direct parent management of child health behavior for the specific purpose of improving child health outcomes (e.g. a planned family health behavior change for the purpose of improving child health such as a parent instituting a family walk after dinner). In adults, when one partner engages in lifestyle change for their own health, positive change is often observed in the other partner [15]. Despite observations that lifestyle changes by one partner in a weight loss program may unintentionally influence the other, the mechanisms and nuances of how this may translate to the impact on the children in the home are unknown. It is not known how a parent's medical weight management journey translates into broader household health behavior modifications, whether by planned health behavior change (e.g. intentionally increased activity), unplanned and unintentional consequences (e.g. a change to the food available in the home based on the parent's grocery choices), or by modeling (e.g. seeing a parent choose more fruits and veggies). The impact on children in this environment is underexplored, particularly when medication management is implemented by the adult guardian of children. With the evidence-based practice of treatment of obesity, including highly effective medications, it is not known how medical treatment of obesity affects household behavioral change. A significant gap exists in understanding the perceptions of family members regarding household health behavior changes when a parent actively participates in their own medical weight management program that includes obesity medication therapy.

## 2 Purpose and objectives of the study

The purpose of the study was to determine the perceptions of the interviewees' participation in the weight loss clinic on the interviewees' children's health behaviors.

## 3 Methods

### 3.1 Study design: This is a qualitative study

Participants: Participants in this study were adult patients in an academic medical weight management clinic. Inclusion criteria included participants had to be prescribed obesity medication. All participants had at least one child, age 18 or younger, in the home. Participants were excluded from the study if they could not speak English or were not taking part in the academic medical weight management clinic.

Procedures: Participants were contacted by phone or email by a member of the research team (PD) to confirm eligibility and review the cover letter consent. The cover letter covered all aspects of the research study including purpose, procedures, risks/benefits, voluntary nature, right to withdraw, confidentiality/data handling, and the use of recording. Verbal consent was obtained after reviewing the cover letter After confirming consent, the telephone interviews were scheduled at the participant's convenience. All interviews were conducted by the same research nurse who has training in qualitative methods and is an experienced qualitative interviewer. The interviews took place in a private office from December 2024 to July 2025. The interviews were audio recorded and transcribed using voice recognition by Zoom. The interviewer subsequently reviewed and edited the transcripts for accuracy against the audio recordings. The participants did not receive any compensation. This study was reviewed and approved by the West Virginia University Institutional Review Board (protocol number 2408022016).

Semi-structured interview guide development: The Family Health Behavior Scale (FHBS), a validated measure consisting of four subscales, was used to inform the interview questionnaire [16]. The FHBS measures family behaviors that may promote overall health and activity. Two senior research team members (TH and PD) created interview questions informed by the FHBS, including mealtime routines, family activities, and parent-child behaviors. An obesity medicine expert then evaluated the questionnaire and provided suggestions for clarity and completeness.

Data Analysis: Analysis was completed using a thematic approach. Transcripts were read and discussed by the research team, and initial codes were identified. Two team members (RS and MP) coded three of the same transcripts, and discrepancies were discussed and resolved with the research team. The remaining transcripts were then coded individually by the same team members and reviewed with the research team. The transcripts were coded in word processing software. These were then organized and discussed, allowing the identification of themes. The themes were discussed amongst the research team to reach a group consensus.

## 4 Results

Ten interviews were completed for the study. The participants were all recruited from the same academic medical weight management clinic. This is a comprehensive multidisciplinary clinic that includes physicians, advanced practice practitioners, dietitians, exercise physiologists, pharmacists, behavioral medicine specialists, and health coaches. All participants in the study, except one, were prescribed a glucagon-like peptide-1 receptor agonist (GLP-1RAs) medication, and all participants took at least one obesity medication. Resulting themes from the study are discussed in more detail below.

### 4.1 Intentional individual dietary choices

The participants described throughout the interviews that they felt more self-aware of the decisions they were making surrounding food. Many of them spoke about the nutrition education that they had received throughout the program and how that changed their view of food, as well as long-term impacts on their nutritional choices.

“The program really helped me to understand the importance of prioritizing protein and making sure to get adequate protein above everything else. And that was something I just had never thought about before.” ∼Participant 3

The majority of the individuals who were interviewed also mentioned they are becoming more aware of the components of the food they are eating, instead of trying to stick to one fad diet plan that has failed for them before.

“Even if that means more calories in a particular food, you know, watching my calories, but being really concerned about the quality of food and making sure that I’m getting the best kind of food into my body, as opposed to, like, diet foods, or specifically low-calorie, low-fat foods, etc.” ∼Participant 3

“The program also was really helpful in me really thinking about making choices about the types of food I’m eating in terms of seeking, less, you know, processed foods and more whole foods.” ∼Participant 3

In addition, several of the participants mentioned that the education they received has impacted their entire family unit, specifically having a positive impact on their children's eating habits.

“I have more healthy choices available, and I force them [my kids] to eat a fruit and a vegetable every day.” ∼Participant 7

“It's not that you need to learn how to cook, because you're a girl, that you need to learn how to cook to feed your body.” ∼Participant 9

### 4.2 Acknowledgement of unintentional dietary changes in families secondary to program involvement

Participants identified changes happening in their household around food and movement since starting the weight management program. While they identified making prioritized decisions to change health behaviors in their household, they also noted changes that happened without a planned effort.

“I'm going to drop you off at drum lessons, and then I've got to go to the gym for an hour and a half. The gym for an hour and a half, and I'll be back. Yeah, what do you need? And then I noticed my son joined track out of nowhere, and, or I'm gonna stop and get a poke bowl, because I need something healthy. Do you want something? And then I noticed that he started making cucumber salads and, and, you know, Asian cucumbers salads and things that he finds to be healthy for his body.” ∼Participant 7

Also, the participants have altered the preparation and storage of food in their household which has also led to unplanned but welcome changes in what foods children choose to each and have access to. They have noted that with a change in their food environment due to their own care plan has led to a change in nutritional intake in kids.

“Yeah, it's more like the going for more like the yogurts and bananas now that I have prepared, yeah, and I cut up the fruit, like this week was watermelon. They’ll go to that then say, hey, I need a bag of chips. Okay, then make popcorn or something like that. They'll go for the fresh fruits now.” ∼Participant 10

In addition, several of the participants have noted a change in perspective about long-term outcomes related to their health, specifically having to do with their family and kids. While some of the changes were done intentionally, there were some outcomes that came about passively, and may have the largest impact on the families. One example of this is that families have started doing meal preparation together and feel this is a positive change however this was not a directed planned or stated outcome within the program

“Yes, they both love to help with it [meal preparation], and they get a lot of satisfaction out of helping with it [meal preparation].” ∼Participant 3

And other have noted that there is an unplanned change their child developing a like for certain foods, such as salad, which they identify as a difference from their own upbringing around food.

“I have a toddler who eats salads, and I was certainly not a toddler who ate salad. And so the fact that she's enthusiastic about that, I think is a real victory of this entire program.” ∼Participant 9

### 4.3 Awareness of health behaviors across individual and family levels

The participants spoke of the behavior changes that had been made as a result of participation in the weight management program. Many had made positive changes to their overall routines, including being more mindful about both diet and exercise changes. As a result, several of the participants noted that these behavior changes had been passed down to the children in the household.

“Structuring meals around protein and healthier foods. It has really been helpful to have those things around my daughter. I have a toddler who eats salads, and I was certainly not a toddler who ate salad. And so the fact that she’s enthusiastic about that, I think is a real victory of this entire program.” ∼Participant 10

“I’m sure that it has, and probably ways that I can never fully see, because things are so different now than they were, but just the sheer volume of how much she sees us eat is very different, and how we portion out meals, and really the priority we place on food. I think the language I use about food and eating and bodies, I think, is very intentional, and hopefully that helps her as well.” ∼Participant 10

Interviewees also noted that the household structure surrounding food and meals had also changed because of participation in the weight loss program. They note positive effects on the family unit, and positive thoughts about how the kids will view food and eating in the future.

“It [food] just was at the forefront of everything I did, and now it is just another thing I do in the day. It is not the central focus of my world. Certainly, I still value dinner time together with my family, and that's a time when we can talk about our days, but it's more about the time together and less about me constantly thinking about the food. ∼Participant 10

“Yes, because my family growing up, we sat in front of the TV to eat. We didn’t go to the dinner table. Everything was pretty much box made, if that made any sense. We had a meat and two starches. Always, it was always, it was always like mashed potatoes and macaroni and cheese. There wasn’t really a lot of vegetables in there. I want him [my kid] to be more diversified.” ∼Participant 5

“We try to pass along education. I know I shared with her about the late-night cravings and how eating breakfast could help that and was a surprise to me. I do think we have some back and forth every once in a while, so a little bit is filtering down to her [my kid] probably, I think so.” ∼Participant 4

Some even note the change in health behaviors, even outside of just diet and exercise, and how that affects the stability and overall attitude of the household.

“They see me as more that they've said that this is the stable home, the calm home, yeah, the consistent home, and they see those behaviors as consistent and then they're joining in.” ∼Participant 7

“They started seeing their mom as a person who put their physical health as a priority, and they started to show interest in their own physical health.” ∼Participant 7

### 4.4 Optimism regarding lifelong changes despite changes surrounding medication coverage

Many of the participants in the study are hopeful that the changes they have made through participation in the weight loss program will allow them to continue living a healthier life regardless of potential loss of insurance coverage for obesity medication. Some note that other medications have been able to help them maintain the weight that they had previously lost.

“But so I'm going to try the naltrexone, Wellbutrin, and you know, hopefully that will help me continue to do what I need to do to get the rest of the way.” ∼Participant 3

Some of the participants note the additional stress that lack of insurance coverage will have on them and how it may hinder the progress they have made through their work in the clinic.

“Wegovy has been just really an excellent thing and has really helped me a lot, and I hate to get off it, but it's just too expensive without any type of insurance coverage.” ∼Participant 3

“I intend to stay on for as long as I can, because I'm very close to my goal. I intend on trying to pay for it out of pocket, but it's a very big hardship for me.” ∼Participant 7

Also, many of the participants noted that the overall weight loss program has really helped change the way they view themselves, food, and their future. Especially, some note that their kids have been a primary source of motivation, saying that they really want to stay healthy in order to be a part of their futures as well.

“My purpose was not to lose weight, to get pretty, it was to be here long term for him.” ∼Participant 5

“My life now compared to what it was, so much better, so much healthier. I feel better about myself. I feel like I could actually live a life that I'm going to be here longer.” ∼Participant 5

Most importantly, one participant notes how impactful the program has been for her, not only by providing medication options, but providing health and diet education. She notes that these changes have even impacted her kids as well and will hopefully lead to a healthier life and better relationship with food for all of them.

“Yes, definitely, because it's working, right? You know, we really want our kids to have healthy relationships with food, and I want to continue to grow in having a healthy relationship with food. So, yes, absolutely.” ∼Participant 3

## 5 Discussion

This qualitative study is exploratory and contributes to a broad understanding of how parent involvement in a comprehensive medical weight management program with the use of obesity medications can affect behavioral factors for others within their household, especially children. Participants identified that their children were exposed to health behavior changes, both from planned parenting efforts and from unplanned impact of the adult's health behavior changes.

The medical weight management program in which the participants were enrolled places emphasis on healthy lifestyle changes while incorporating obesity medications, and participants did report purposeful changes to their own, and their child's, dietary choices based on these interventions (e.g. insisting that a child eat one fruit and one vegetable each day). Participants also identified that they are now more mindful about both their individual health behavior changes and also the health behavior changes of their families.

However, participants also reported unplanned dietary changes that their house made, which they also attributed to their involvement in the program, such as modeling eating smaller portions. Finally, they had optimism that these changes can be lifelong and benefit them and their family members despite changes in medication coverage.

Information about lifestyle changes can be found everywhere: on the internet, billboards, social circles, etc. However, one of the most important places these discussions can occur is in a healthcare practitioner's office during a routine wellness visit. Discussions about exercise, lifestyle changes, or weight loss are not always easy to navigate in the healthcare setting and often do not happen [17]. However, these discussions can have an impact on patients' implementation of health behavior changes. Previous studies show that alteration of lifestyle habits are more likely to occur if recommendations are made by a healthcare professional whom patients can trust [18]. The most important part about initiating these conversations is to do so after establishing mutual trust and understanding. Also, patients are more responsive to the discussion and engage in the change of behavior when they are an active part of the planning process [19]. Though adding on medications that change the innate drives for energy intake may have an additional impact on instituting health behavior change in families.

A relatively new addition to treating obesity in the clinical setting is the use of effective obesity medications, such as GLP-1RAs [20,21] and other approved oral obesity medication options. Specifically, the GLP-1RA class of obesity medication has well-established effects on appetite suppression and satiety through biological and hormonal pathways; however, emerging studies have also demonstrated a link between this class of obesity medications and certain food-related behaviors and dietary preferences [22]. While on GLP-1RAs, patients have been shown to reduce total energy consumption by reducing meal portions and decreased preference toward calorically dense foods [23]. Studies have shown that patients are more likely to report improved control of food cravings and ingestive/consummatory behaviors while on GLP-1RAs [24]. If GLP-1Ras and other obesity medications positively impact parent health behavior change, such as via modeling smaller portion sizes or buying less calorically dense foods for the household, and parent health behavior change does, in fact, positively impact child health behaviors, the downstream positive implications of GLP-1RA and other obesity prescription should be considered.

### 5.1 Limitations

This study was not without limitations. The study is exploratory and future prospective studies would be needed to identify causation of program elements to measurable outcomes. The study did have a low sample size; however, thematic saturation was reached after ten participants. This is in keeping with previous work showing that saturation is often reached between 9 and 17 interviews [25]. The interview guide was created from a well-established survey, the FHBS, which could have driven the lower number of interviews needed through a narrower focus to the interview guide. Also, the principal investigator (PI) of the study is a physician in the program from which patients were recruited, which can introduce social desirability bias. To mitigate this, the PI did not actively recruit patients to the study; recruitment was conducted through patient portal methods, and a trained research nurse (PD), who is not part of the clinical setting, completed the interviews and de-identified the transcripts prior to distributing them to the research team. Selection bias is also a concern as all patients in this study are taking part in a comprehensive weight management program which may change motivation of the participant to take part in lifestyle change and therefore outcomes of their interviews.

## 6 Conclusion

The rise in incidence of childhood overweight and obesity has been a growing concern in the United States as well as on a global scale. While significant evidence exists demonstrating the benefit of family-based behavioral interventions on child weight outcomes, we sought to fill the gap in exploring how parent participation in a medical weight management clinic trickles down to affect behavioral change in their children. Utilizing evidence based qualitative patient interviews, participants demonstrated both conscious and unconscious behavioral changes and identified positive behavioral change in their children. Additionally, the participants expressed optimism that these behavioral changes will persist in the face of potential changes to insurance coverage of obesity medications, demonstrating the long-standing benefits of healthy lifestyle changes on dietary choices and weight outcomes.

Key takeaways from this study:

• Parent efforts to improve their own health behaviors can consciously and unconsciously result in efforts to improve child health behaviors.
• Further research is needed to further understand the processes by which health behavior transmission impacts childhood health and wellbeing.
• It is possible that future efforts to advocate for access to adult weight management treatment should include a recognition of the possible downstream benefits of improving overall household health.

## Author contribution, such as via the CrediT format

The concept of the submission was by TH and JL. Methodology and Formal Analysis were completed by TH and PD. MP and RS performed data curation. TH provided Supervision and Project Management. TH, JL, MP and RS wrote the first draft. TH, JL, MP, RS, and PD all reviewed and edited subsequent drafts and approved the final submission and publication.

## Disclosures

Treah Haggerty is an editor on the Obesity Pillars board. The authors declare no other conflicts of interest.

## Ethical adherence and ethical review

The study was approved by the West Virginia University Institutional Review Board: 2408022016. Responsibility for editorial decisions and peer review process for this article was delegated to non-author Editors or non-author Associate Editors.

## Declaration of Artificial Intelligence (AI) and AI-assisted technologies

During the preparation of this work the author(s) did not use AI.

## Source of funding

This research did not receive any specific grant funding, including that from funding agencies in the public, commercial, or not-for-profit sectors.
